# Antimicrobial Resistance and Molecular Characteristics in Tigecycline-Resistant *Escherichia coli* Isolates from Broilers in Jinan City, China

**DOI:** 10.3390/microorganisms14061264

**Published:** 2026-06-04

**Authors:** Rong Xi, Ban Li, Yunchen Zhou, Chengbo Wen, Yue Wu, Zhiyong Wu, Dexian Zhang, Jichang Li

**Affiliations:** 1 School of Animal Science and Technology, Foshan University, Foshan 528231, China; 17866927857@163.com (R.X.); a13138288071@163.com (B.L.); zhouyunchen2025@126.com (Y.Z.); bwcheng0023@163.com (C.W.); wuyue12262022@163.com (Y.W.); 2 College of Veterinary Medicine, Northeast Agricultural University, Harbin 150030, China; wuzhiyong@neau.edu.cn

**Keywords:** *E. coli*, tigecycline resistance, virulence genes, *tet*(X4), MLST

## Abstract

The emergence of plasmid-mediated tigecycline resistance in food-producing animals poses a significant threat to global public health by compromising a last-resort antimicrobial. This study investigated the prevalence, antimicrobial susceptibility profiles, and molecular genetic characteristics of tigecycline-resistant *Escherichia coli* (*E. coli*) isolated from broiler cecal samples in the Jinan region of Shandong Province, China, between 2020 and 2024. Antimicrobial susceptibility testing showed that high-level resistance to colistin (87.5%, 14/16), florfenicol (81.3%, 13/15), and enrofloxacin (75.0%, 12/16) was observed. Notably, a single isolate was resistant to meropenem (6.3%, 1/16). Whole-genome sequencing and subsequent in silico analysis demonstrated that all 16 tigecycline-resistant isolates harbored the tigecycline resistance gene *tet*(X4), the primary determinant of resistance. Molecular typing identified a diverse population structure, with sequence type 224 (ST224) being the dominant clone, accounting for 37.5% (6/16) of the isolates. The genetic milieu of resistance was complex, characterized by the co-existence of *tet*(X4) with multiple other clinically important resistance genes, including the mobile colistin resistance gene *mcr*-1 (87.5%, 14/16) and various extended-spectrum β-lactamase genes such as *bla*CTX-M variants and, critically, *bla*NDM-5. Furthermore, an array of virulence factor genes was identified, with a particularly high prevalence of the toxin gene *astA* (68.8%, 11/16) and the bacteriocin gene *cma* (50.0%, 8/16), indicating pathogenic potential. This convergence of resistance and virulence in a foodborne pathogen highlights an urgent need for continuous One Health surveillance to mitigate the risks posed by these potentially untreatable “superbugs” to both animal and human health.

## 1. Introduction

The discovery of antibacterial agents constitutes a seminal milestone in the history of human medicine. However, their widespread and frequently irrational use has progressively intensified the challenge of bacterial resistance [1]. As the world’s leading producer of livestock and poultry, China’s animal husbandry sector consumed nearly 100,000 tons of antimicrobials annually prior to the implementation of policies aimed at reducing and prohibiting antibiotic use [2]. Tetracyclines and florfenicol were the most extensively administered classes, a practice that markedly accelerated the emergence of bacterial resistance in food-producing animals. The development of antimicrobial resistance (AMR) in animal-derived bacteria not only threatens the sustainability of the livestock industry but also poses a substantial risk to public health.

Tigecycline, a third-generation tetracycline derivative, exerts its antibacterial activity by binding to the 16S rRNA of the bacterial 30S ribosomal subunit [3]. Tigecycline exhibits a broad spectrum of activity and retains potent efficacy against numerous drug-resistant pathogens, including methicillin-resistant *Staphylococcus aureus* (MRSA), vancomycin-resistant *Enterococci* (VRE), extended-spectrum β-lactamase (ESBL)-producing *Escherichia coli* (*E. coli*), and carbapenem-resistant *Acinetobacter baumannii*. Given its robust efficacy against multidrug-resistant (MDR) organisms, tigecycline is widely regarded as a “last-resort” antibiotic for the treatment of infections caused by MDR bacteria. Although approved for clinical use in China in 2012, its application is strictly confined to human medicine and remains explicitly prohibited in livestock and poultry production [4].

Despite its capacity to circumvent classical tetracycline resistance mechanisms, the escalating use of tetracyclines across multiple sectors and the expanded clinical deployment of tigecycline have fostered the emergence of resistance to this last-resort agent [5]. The discovery of plasmid-mediated high-level tigecycline resistance marked a critical turning point: tigecycline-resistant *E. coli* was first recovered from a porcine sample in 2019, leading to the designation of the resistance-conferring gene as *tet*(X4) [6]. Subsequently, *E. coli* strains exhibiting high-level tigecycline resistance were isolated from shrimp samples in 2020 [7]. Since these initial discoveries, tigecycline-resistant *E. coli* has been successively detected in porcine hosts in China [8], chicken in South Asia [9], and bovine hosts in Europe [10], as well as in retail meat products in China [11]. Furthermore, such resistant strains have been documented between 2020 and 2023 in multiple countries across diverse continents, including the United States, Egypt, Pakistan, and Côte d’Ivoire [12], underscoring the global dimension of this threat.

Parallel to this veterinary and food-chain dissemination, the clinical efficacy of tigecycline has also been increasingly compromised, posing a significant public health threat in China. Since its introduction, clinical reports of tigecycline-resistant “superbugs” have accumulated, and recent multicenter studies reveal that tigecycline-resistant *Enterobacteriaceae* strains in Chinese hospitals often exhibit multidrug resistance, with *tet*(A) mutations being a prevalent mechanism [11,13]. A particularly alarming development bridging clinical and environmental reservoirs is the identification of a 193 kb high-risk hybrid plasmid (p193k-tetX4) that harbors *tet*(X4) alongside other critical resistance genes like *mcr* and *bla*NDM [14]. This plasmid family has demonstrated extensive cross-host and cross-border dissemination among humans, animals, and the environment, with a dominant lineage circulating widely in China and beyond. The convergence of clinical resistance mechanisms and the rapid spread of transferable resistance genes such as *tet*(X4) across various ecosystems underscores the urgent need for coordinated “One Health” surveillance to safeguard the utility of tigecycline.

The widespread dissemination of tigecycline-resistant *E. coli* throughout the livestock and poultry production chain consequently poses a grave threat to both the horizontal transfer of antimicrobial resistance genes and public health security. Systematic surveillance of resistance patterns and in-depth investigation into the underlying resistance mechanisms of tigecycline-resistant *E. coli* are therefore of paramount importance. Against this backdrop, the present study investigated the antimicrobial resistance profiles of tigecycline-resistant *E. coli* isolates recovered from a broiler slaughterhouse in Jinan City, China, between 2020 and 2024. The current resistance status and key molecular genetic characteristics of these isolates were analyzed to provide a scientific basis for effectively curbing the dissemination of resistance determinants and guiding the prudent use of antimicrobials in livestock farming.

## 2. Materials and Methods

### 2.1. Samples and Isolates

A total of 600 cecal content samples were collected from five broiler slaughterhouses in Jinan, Shandong Province, China, between 2020 and 2024. Sampling was conducted at approximately equal intervals across the study period, with 120 samples obtained from each slaughterhouse. Cecal contents were aseptically collected immediately after evisceration from randomly selected healthy broiler carcasses on the slaughter line. Each sample (approximately 5 g) was placed into a sterile 50 mL centrifuge tube, transported to the laboratory on ice within 4 h of collection, and processed within 24 h. For *E. coli* isolation, approximately 1 g of each cecal content sample was enriched in 9 mL of buffered peptone water (Sigma-Aldrich, Shanghai, China) and incubated at 37 °C for 24 h. A loopful of the enriched culture was then streaked onto MacConkey agar (Aobox, Beijing, China) plates and incubated at 37 °C for 24 h. Presumptive *E. coli* colonies (lactose-fermenting, pink colonies) were picked, and identified using a Bruker MALDI Biotyper system (Bruker Daltonik, Bremen, Germany) with the MBT Compass Library (Revision D) on a Microflex LT mass spectrometer (Bruker Daltonics, Bremen, Germany).

### 2.2. Antimicrobial Susceptibility Testing

The minimum inhibitory concentrations (MICs) of tigecycline against *E. coli* isolates were determined using the broth microdilution method, with *E. coli* ATCC 25922 serving as the quality control strain. Each isolate was tested in triplicate. Tigecycline-resistant isolates were subjected to further antimicrobial susceptibility testing. Results were interpreted according to the breakpoints recommended by the European Committee on Antimicrobial Susceptibility Testing (EUCAST, Version 12.0); the clinical breakpoint for tigecycline is >0.5 mg/L. Antimicrobial agents used in this study were purchased from MeilunBio (Dalian, China) (Table 1).

**Table 1:** Breakpoints for antimicrobial susceptibility testing in this study.

Antimicrobial Agents	Susceptible	Mediate	Resistant	Range (μg/mL)
Tigecycline	≤0.5	-	>0.5	0.06–64
Meropenem	≤1	2	≥4	0.06–64
Enrofloxacin	≤0.5	1–2	≥4	0.03–32
Colistin	-	≤2	≥4	0.06–64
Kanamycin	≤16	32	≥64	0.25–256
Gentamicin	≤2	4	≥8	0.06–64
Ceftiofur	≤2	4	≥8	0.25–256
Florfenicol	≤2	4	≥8	0.25–256

### 2.3. Genomic DNA Extraction of Tigecycline-Resistant E. coli

Purified Tigecycline-resistant *E. coli* isolates were reactivated and inoculated into 5 mL of LB broth (Oxoid, Shanghai, China), followed by incubation at 37 °C for 18 h. Genomic DNA was extracted from the bacterial cultures using a bacterial genomic DNA extraction and purification kit (TIANGEN, Beijing, China) according to the manufacturer’s instructions.

### 2.4. MLST Typing, Serotyping, and of Tigecycline-Resistant E. coli

Sequencing was performed using an Illumina Nextera XT library with 2 × 300 bp paired-end reads (BGI, Shenzhen, China), yielding an average of 2,232,425 reads per isolate (63× average coverage). Raw data were assembled using SPAdes (version 3.0). Multilocus sequence types (MLST), plasmid replicon types, serotypes, virulence genes, and antimicrobial resistance genes were identified using MLST 2.0, PlasmidFinder 2.0, SeroTyperFinder 2.0 and ResFinder 3.0, respectively, all available from the Center for Genomic Epidemiology database (http://genomicepidemiology.org/, accessed on 15 March 2026). Plasmids were also analyzed using PLACNETw (https://castillo.dicom.unican.es/upload/, accessed on 15 March 2026).

Parsnp v2.0 was used to align the core genome of ESBL-producing *E. coli* isolates, to call single nucleotide polymorphisms (SNPs), and to generate a core-genome SNP tree with 1000 bootstrap resamples [15].

### 2.5. Virulence Gene Distribution of Tigecycline-Resistant E. coli

VirulenceFinder 2.0 available from the Center for Genomic Epidemiology database (http://genomicepidemiology.org/, accessed on 20 March 2026) was hired to detect the virulence gene distribution of Tigecycline-resistant *E. coli*. A panel of virulence-associated genes was selected based on their established roles in the pathogenesis of extraintestinal pathogenic *E. coli* (ExPEC) and their prevalence in animal-derived strains. These genes encompass four functional categories critical for adhesion (*fimH*, *fdeC*, *lpfA*, *yeh*), iron acquisition (*irp2*, terC, *iroN*, *fyuA*), enzyme (*gad*), Stress tolerance (*htrA*), and toxin production (*hlyE*, *cma*, *astA*, *hha*) [16]. Screening these factors aimed to characterize the pathogenic potential of tigecycline-resistant *E. coli* and explore possible associations between resistance and virulence.

## 3. Results

### 3.1. Isolation and Identification of E. coli

A total of 584 presumptive *E. coli* isolates were recovered from the 600 cecal content samples based on characteristic colony morphology on MacConkey agar. Following MALDI-TOF MS confirmation, 537 *E. coli* strains were identified as *E. coli*, yielding a sample-level isolation rate of 89.5% (537/600). The remaining isolates were identified as non-*E. coli* species and were excluded from subsequent analyses. Among these 537 isolates, 16 (3.0%) were subsequently confirmed as Tigecycline-resistant according to results of antimicrobial susceptible testing.

### 3.2. Antimicrobial Resistance of Tigecycline-Resistant E. coli Isolates

Antimicrobial susceptibility testing revealed that among the 16 Tigecycline-resistant *E. coli* isolates, resistance to colistin was most prevalent (87.5%, 14/16). High resistance rates were also observed for florfenicol (81.3%, 13/16), enrofloxacin (75.0%, 12/16), kanamycin (68.8%, 11/16), ceftiofur (62.5%, 10/16), and gentamicin (43.8%, 7/16). In contrast, only one isolate was resistant to meropenem (6.3%, 1/16). The resistance rate for each antimicrobial was defined as the number of resistant isolates divided by the total number of Tigecycline-resistant *E. coli* isolates (n = 16).

### 3.3. Characteristics of tet(X4) Gene-Carrying Plasmids

Bioinformatic analysis predicted that all *tet*(X4) genes were associated with plasmid-derived contigs, which ranged in size from approximately 43 to 152 kb. Based on in silico prediction, putative plasmid replicon types identified among contigs harboring *tet*(X4) genes included IncX1 (4/16), IncFIA(HI1) (3/16), IncHI1A (3/16), IncHI1B (2/16), IncFIA_I1 (2/16), IncFII_1 (1/16), and IncFIA-IncHI1B (1/16). Additionally, these *tet*(X4)-carrying plasmid-derived contigs were predicted to carry additional antimicrobial resistance genes, including those conferring resistance to chloramphenicol (*floR*), sulfonamides (*sul1* and *sul3*), and trimethoprim (*dfrA1* and *dfrA12*).

### 3.4. MLST Typing, Antimicrobial Resistance Genes, and Virulence Factor Genes of Tigecycline-Resistant E. coli

**Figure 1:**
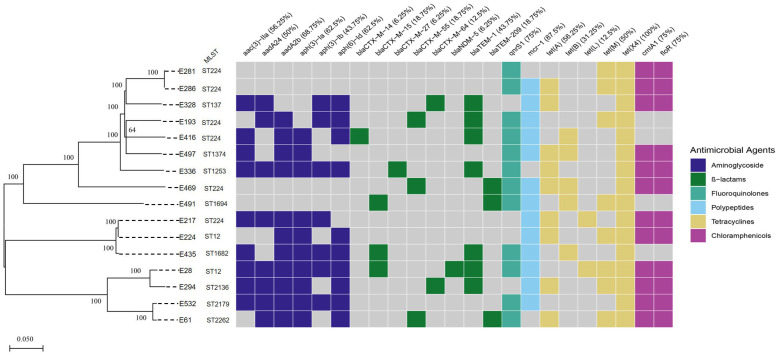
Molecular epidemic characteristics of 16 Tigecycline-resistant *E. coli* isolates. Bar, 0.050 substitutions per nucleotide position.

The MLST typing results of the Tigecycline-resistant *E. coli* isolates revealed diverse sequence types (STs), as shown in Figure 1. Among these, ST224 was the most prevalent, accounting for 37.5% (6/16) of the isolates, followed by ST12, which was detected in 12.5% (2/16) of the isolates. ST37, ST1374, ST1253, ST1694, ST1682, ST2136, ST2179, and ST2262 were each identified in a single isolate.

The tigecycline-resistant *E. coli* isolates carried a diverse array of antimicrobial resistance genes (Figure 1). Notably, the *tet*(X4) gene cluster was universally present across all isolates, with a RamR A19V substitution detected in approximately one-third of the strains. High carriage rates (≥75%) were observed for *mcr-1*, *cmlA1*, *floR*, and *qnrS1*. Various aminoglycoside and β-lactamase resistance determinants were also identified, with *aph(3)-Ia*, *aph(6)-Ib*, *aadA2b*, and *bla*TEM-1 being the most prevalent among their respective classes. Additionally, quinolone resistance-associated mutations, including GyrA (S83L, D87N), ParC (S80I), and ParE (S458A), were detected.

**Figure 2:**
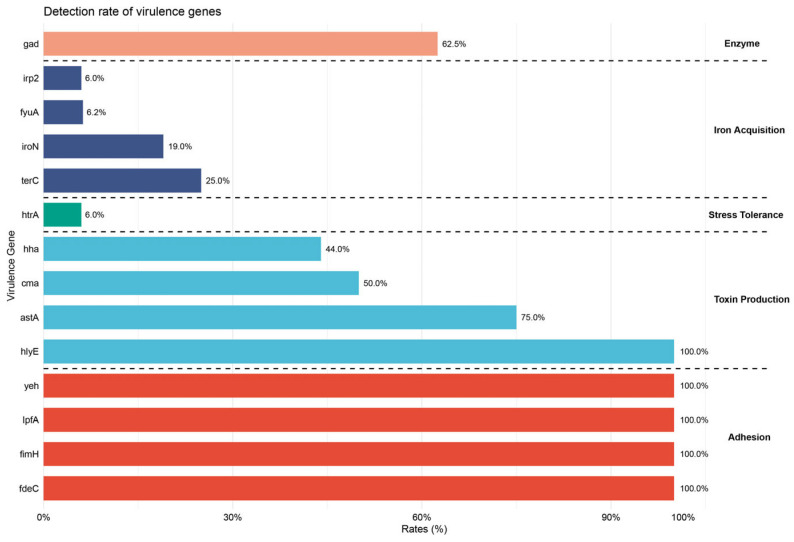
Virulence gene distributions among 16 Tigecycline-resistance *E. coli* isolates.

The tigecycline-resistant *E. coli* isolates also carried multiple virulence factor genes (Figure 2). All strains harbored *fdeC*, *fimH*, *lpfA*, *yeh*, and *hlyE*. Among the remaining virulence-associated genes, *astA* showed the highest prevalence (approaching 70%), followed by *cma* (present in half of the isolates). Other genes, including *irp2*, *terC*, *hlyA*, *iroN*, and *fyuA*, were detected at lower frequencies, ranging from approximately 30% to below 10%.

## 4. Discussion

In this study, we investigated the prevalence, antimicrobial susceptibility, and molecular characteristics of Tigecycline-resistant *E. coli* isolated from broiler cecal samples in the Jinan region. The overall *E. coli* isolation rate of 89.5% is consistent with the high carriage rates commonly reported in poultry, which serve as a significant reservoir for commensal and pathogenic strains [17,18]. The emergence of 16 Tigecycline-resistant isolates, all carrying the *tet*(X4) gene, is of particular concern. This finding corroborates recent reports on the increasing detection of plasmid-mediated tigecycline resistance in food-producing animals in China, highlighting the rapid dissemination of this novel resistance mechanism [6,19].

Among the 537 *E. coli* strains isolated from cecal content samples, 16 (3.0%) were confirmed as tigecycline-resistant. This detection rate is notably higher than the 0.65% prevalence reported in a recent study from Yangzhou, China, which screened 618 *E. coli* isolates from diverse sources including patients, pigs, chickens, and vegetables [20]. The higher prevalence observed in our study may reflect the focused sampling within a poultry slaughterhouse setting, where selective pressures from antimicrobial use in livestock production may facilitate the enrichment of resistant strains. The recovery of tigecycline-resistant *E. coli* from broiler cecal samples is consistent with previous reports documenting the widespread occurrence of *tet*(X4)-positive strains in food-producing animals across China [19].

In our study, bioinformatic analysis predicted that all *tet*(X4) genes were located on plasmid-derived contigs ranging from approximately 43 to 152 kb. The identified plasmid replicon types included IncX1 (4/16), IncFIA(HI1) (3/16), IncHI1A (3/16), IncHI1B (2/16), and others. This diversity of plasmid backbones is consistent with the growing body of literature documenting the presence of *tet*(X4) on multiple plasmid incompatibility groups, including IncX1, IncFIB, IncFIA, and IncHI1 [21,22]. Notably, IncX1 plasmids have been identified as key vectors for *tet*(X4) dissemination across different *E. coli* hosts and geographical regions [23]. The association of *tet*(X4) with mobile elements such as ISCR2, which is known to facilitate the transposition of adjacent DNA sequences through rolling-circle replication, further enhances the mobility of this resistance gene [24].

The molecular typing data revealed a diverse population structure among the Tigecycline-resistant isolates, with ST224 being the dominant sequence type. The predominance of ST224, which has been previously associated with MDR phenotypes in animals, suggests its potential role as a successful epidemic clone in poultry [25]. The diversity of STs, including ST12, ST37, and others, indicates that the *tet*(X4) gene is not restricted to a single genetic background but is rather spreading horizontally among different *E. coli* lineages [23]. This horizontal gene transfer is likely mediated by mobile genetic elements that frequently carry multiple resistance genes, as evidenced by the extensive resistance profiles observed [26,27].

Furthermore, the isolates harbored a considerable array of virulence factor genes, and the high prevalence of enteroaggregative *E. coli* heat-stable enterotoxin 1 gene (*astA*, 68.8%) and colicin M gene (*cma*, 50.0%) among Tigecycline-resistant *E. coli* is noteworthy. The presence of iron acquisition systems (*irp2*, *fyuA*, *iroN*) and toxins (*hlyA*) in a subset of isolates indicates their potential to cause extra-intestinal infections in humans. The combination of a highly virulent genetic makeup with an MDR profile in a dominant food-animal clone like ST224 constitutes a substantial threat to both animal health and food safety.

## 5. Conclusions

In conclusion, this study reveals a high prevalence of *tet*(X4)-mediated tigecycline resistance in *E. coli* from broilers in the Jinan region. These isolates are commonly MDR, co-harboring genes conferring resistance to last-resort drugs like polymyxins and carbapenems, and also carry multiple virulence factors. The clonal expansion of ST224 is a key driver of this dissemination. Continuous surveillance of antimicrobial resistance in animal reservoirs is imperative to understand and mitigate the public health risks posed by these pathogens. Effective control measures should be implemented to limit the spread of MDR bacteria in livestock populations, including the targeted depopulation or isolation of colonized animal flocks to prevent onward transmission. Furthermore, strict adherence to on-farm biosecurity protocols is essential to reduce environmental contamination and interrupt transmission pathways within and between production facilities, such as thorough cleaning and disinfection of animal housing, equipment, and transport vehicles.

## Data Availability

The data presented in this study are openly available in GenBank at NCBI, reference number PRJNA1464078.
